# Impact of carbendazim on cellular growth, defence system and plant growth promoting traits of *Priestia megaterium* ANCB-12 isolated from sugarcane rhizosphere

**DOI:** 10.3389/fmicb.2022.1005942

**Published:** 2022-12-20

**Authors:** Anjney Sharma, Xiu-Peng Song, Rajesh Kumar Singh, Anukool Vaishnav, Saurabh Gupta, Pratiksha Singh, Dao-Jun Guo, Krishan K. Verma, Yang-Rui Li

**Affiliations:** ^1^Key Laboratory of Sugarcane Biotechnology and Genetic Improvement (Guangxi), Ministry of Agriculture, Sugarcane Research Center, Chinese Academy of Agricultural Sciences, Guangxi Academy of Agricultural Sciences (GXAAS), Nanning, Guangxi, China; ^2^Guangxi Key Laboratory of Sugarcane Genetic Improvement, Sugarcane Research Institute, Guangxi Academy of Agricultural Sciences (GXAAS), Nanning, Guangxi, China; ^3^Department of Biotechnology, GLA University, Mathura, UP, India; ^4^State Key Laboratory for Conservation and Utilization of Subtropical Agro-Bioresources, College of Agriculture, Guangxi University, Nanning, Guangxi, China

**Keywords:** carbendazim, PGPR, CLSM, reactive oxygen species, oxidative stress, antioxidant enzymes

## Abstract

Agrochemicals are consistently used in agricultural practices to protect plants from pathogens and ensure high crop production. However, their overconsumption and irregular use cause adverse impacts on soil flora and non-target beneficial microorganisms, ultimately causing a hazard to the ecosystem. Taking this into account, the present study was conducted to determine the high dosage of fungicide (carbendazim: CBZM) effects on the rhizobacteria survival, plant growth promoting trait and reactive oxygen species (ROS) scavenging antioxidant enzyme system. Thus, a multifarious plant growth promoting rhizobacteria (PGPR) isolate, ANCB-12, was obtained from the sugarcane rhizosphere through an enrichment technique. The taxonomic position of the isolated rhizobacteria was confirmed through 16S rRNA gene sequencing analysis as *Priestia megaterium* ANCB-12 (accession no. ON878101). Results showed that increasing concentrations of fungicide showed adverse effects on rhizobacterial cell growth and survival. In addition, cell visualization under a confocal laser scanning microscope (CLSM) revealed more oxidative stress damage in the form of ROS generation and cell membrane permeability. Furthermore, the increasing dose of CBZM gradually decreased the plant growth promoting activities of the rhizobacteria ANCB-12. For example, CBZM at a maximum 3,000 μg/ml concentration decreases the indole acetic acid (IAA) production by 91.6%, ACC deaminase by 92.3%, and siderophore production by 94.1%, respectively. Similarly, higher dose of fungicide enhanced the ROS toxicity by significantly (*p* < 0.05) modulating the stress-related antioxidant enzymatic biomarkers in *P. megaterium* ANCB-12. At a maximum 3,000 μg/ml CBZM concentration, the activity of superoxide dismutase (SOD) declined by 82.3%, catalase (CAT) by 61.4%, glutathione peroxidase (GPX) by 76.1%, and glutathione reductase (GR) by 84.8%, respectively. The results of this study showed that higher doses of the fungicide carbendazim are toxic to the cells of plant-beneficial rhizobacteria. This suggests that a recommended dose of fungicide should be made to lessen its harmful effects.

## Introduction

Sugarcane (*Saccharum officinarum* L.) is a commercially important crystal sugar and bioenergy-producing cash crop grown in tropical and subtropical regions around the world. China is the world’s third largest sugarcane producer, which contributes to the economic prosperity of indigenous farmers (Sharma et al., 2021). However, sugarcane productivity was hampered due to diverse pathogen attacks. Among them, Pokkahboeng caused by *Fusarium verticillioides*, wilt caused by *Fusarium sacchari*, red rot caused by *Colletotrichum falcatum*, smut caused by *Sporisorium scitamineum*, and twisted leaves caused by *Phoma* sp. are re-emerging diseases of sugarcane. In China, these fungal diseases cause serious yield losses (about 5–50%) in commercial sugarcane production (Liu et al., 2017; Xu et al., 2019; Hossain et al., 2020). Most of these diseases of sugarcane have been associated with several other fungal diseases. To control the negative effects of these diseases, application of fungicides, particularly carbendazim (methyl 1H-benzimidazol-2-yl carbamate, C_9_H_9_N_3_O_2_), a broad-spectrum fungicide, has been widely used to protect sugarcane and other crops from fungal diseases (Shao and Zhang, 2017). Carbendazim (CBZM) has a huge global market worth over $200 million at the user level. Although some credit goes to CBZM application, the continuous application of fungicide reduces the affinity of this chemical fungicide as well as, through various mechanisms, the resistance also developed in targeted plant pathogens (Ma and Michailides, 2005; Martinez-Rossi et al., 2008). A field survey recently found approximately 15% of the CBZM-resistant *Fusarium* species complex in a chewing cane field in China (Xu et al., 2019). Due to insufficient knowledge and understanding of disease control, apart from the recommended dose (i.e., 0.1% CBZM), farmers extensive use the fungicide, resulted in serious health implications for soil fertility, non-targeted soil beneficial microorganisms, agriculture, the environment, water, air, and animal health (Pankaj et al., 2016a,b; Sharma et al., 2016; Xu et al., 2019; Bhatt et al., 2021). There is now overwhelming evidence that due to their long half-life (up to 12 months) and their relatively high stability, some of these organic compounds (OC) are classified as hazardous xenobiotics by the WHO (World Health Organization), which imposes a potential risk and could pollute every life form on the earth (Zhang et al., 2013; Bhatt et al., 2019; Mishra et al., 2021).

In an agriculture system, plants rely on beneficial soil microorganisms’ processes, which include nutrient cycling and decomposition of organic matter. Any loss of soil microbial flora due to fungicide application could result in deteriorating soil health and lead to reduced field productivity. The repetitive use of fungicides or pesticides increases their persistence in the soil, which leads to affected microbial growth and performance (Gill and Garg, 2014; Al-Enazi et al., 2022). Reports are available highlighting the dose dependent effects of fungicides on plant growth promoting rhizobacteria (PGPR), i.e., with the increase of dose adverse effects of fungicide/pesticide on PGPR (Yang et al., 2011; Mundi et al., 2020). Recently, Shahid et al. (2021a) reported that treatments of various pesticides adversely affect the growth, morphology, viability, cellular respiration, EPS, and biofilm formation, as well as plant growth promoting activities, in different bacterial species. In addition, pesticides also induce oxidative stress by generating ROS (reactive oxygen species). ROS molecules, *viz.*, hydroxyl radical (^–^OH), hydrogen peroxide (H_2_O_2_), superoxide radical (O_2_^–^), etc., lead to lipid peroxidation, protein denaturation, enzyme inactivation, metabolic disturbance, and alteration of the genetic materials, which finally affects the cellular integrity (Bernat et al., 2018; Shahid et al., 2021b). Thus, to neutralize the toxic effects of ROS molecules, living organisms produce antioxidant enzymatic machinery that is needed to evaluate the survival mechanisms of microbes. In addition, such a type of study is a pre-requisite to amending pesticide regulations in the near future.

Keeping in view the problem of fungicide toxicity to soil-beneficial plant growth promoting bacteria, the present study was intended to determine carbendazim toxicity on sugarcane rhizobacterial physiology, plant growth-promoting (PGP) activity, and the antioxidant enzyme defense system.

## Materials and methods

### Soil sampling, enrichment, and isolation of rhizobacteria

In the present study, randomly selected a total of five rhizosphere soil samples were collected from the fungicide applied sugarcane field near the Sugarcane Research Institute, Guangxi Academy of Agricultural Sciences, Nanning, China. From collected rhizospheric soil samples, stone, soil debris, and plant parts were removed and subjected to shade drying for 24–48 h. The composite sieved rhizospheric soil sample was enriched in mineral salt medium (MSM) amended with 500 μg/ml of CBZM (50% w/v of the active ingredient) for 96 h for the isolation of fungicide tolerant bacteria. After incubation of the fungicide enriched soil sample, the bacteria isolation was carried out by the serial dilution method on mineral salt agar plates supplemented with 500 μg/ml CBZM and incubated for 72–96 h at 28°C. After the incubation, a total of 18 dominant bacterial colonies (ANCB-1 to ANCB-18) were screened and conserved in 50% glycerol at −80°C for further use.

### Intrinsic tolerance of isolated rhizobacteria against carbendazim

The intrinsic fungicide tolerance ability of the obtained 18 rhizobacterial isolates was determined through minimal salt agar (MSA) medium (g/L: KH_2_PO_4_ 1.0 g, K_2_HPO_4_ 1.0 g, NH_4_NO_3_ 1.0 g, MgSO_4_.7H_2_O 0.2 g, FeSO_4_.7H_2_O 0.01 g, CaCl_2_.2H_2_O 0.02 g, agar powder 18.0 g, and pH 6.5). Briefly, actively growing fresh rhizobacterial cultures (10^8^ cells/ml) were spot inoculated on MSA plates containing varying concentration of fungicide CBZM, *viz.*, 500, 1,000, 1,500, 2,000, 2,500, and 3,000 μg/ml. After the inoculation, all the inoculated medium plates were incubated at 28 ± 2°C for 48–72 h. Followed by highest fungicide, tolerant rhizobacteria was selected for further study.

### Morphological, biochemical, and metabolic characterization of the rhizobacteria

The selected bacteria isolate ANCB-12 showed a higher level of CBZM tolerance was morphologically and biochemically characterized by following Bergey’s Manual of Determinative Bacteriology (Tindall et al., 2010). Furthermore, isolate ANCB-12 was metabolically characterized by carbon source utilization pattern using BIOLOG GNIII MicroPlate™ (Biolog, Inc., Hayward, CA, USA) following the manufacturer’s instruction.

### Molecular identification by phylogenetic analysis

By adopting the standard protocol of Pospiech and Neumann (1995), genomic DNA was extracted from the actively grown rhizobacterial culture. To amplify the 16S rRNA gene, the universal 16S rRNA gene primers PA (5′-AGA GTT TGA TCC TGG CTC AG-3′) and PH (5′-AAG GAG GTG ATC CAG CCG CA-3′) were used (Sharma et al., 2015). The 50 μl PCR reaction mixture contains 50 ng of DNA template, 10× reaction buffer, 2.5 mM dNTPs, 10 pM of each primer, and 3 U of *Taq* polymerase. PCR condition in the thermal cycler was as follows: at 94°C initial denaturations for 4 min, followed by denaturation at 94°C for 45 s of 35 cycles, annealing at 51°C for 1.5 min, and at 72°C elongations for 90 s, and 72°C final extensions for 7 min. The Amplified 16S rRNA gene PCR product was purified using the BioFlux PCR purification kit (China) by following the manufacturer’s protocol. The purified PCR product was sequenced and analyzed for percent (%) similarities with the available sequences in the GenBank database at the NCBI website. The nucleotide sequence of the bacteria ANCB-12 was deposited in the GenBank sequence database (ON878101). Based on evolutionary distances and Jukes–Cantor coefficient calculation, a neighbor-joining (NJ) phylogenetic tree was constructed through MEGA-X software (Sharma et al., 2019).

### Growth kinetics study of selected isolate

Among the all tested bacterial isolates, isolate ANCB-12 showed the highest tolerance of carbendazim and was selected for growth kinetic study. The selected isolate was inoculated in liquid MSM containing 0, 500, 1,000, 2,000, and 3,000 μg/ml concentration of CBZM and incubated at 150 rpm for 60 h at 28 ± 2°C. To make the growth kinetic curve, the optical density at 600 nm was taken every 12 h.

### Cell viability under carbendazim stress

Cell viability in terms of CFU count was carried out by spreading the 100 μl aliquots of 48 h grown culture of each CBZM stress level. CFU was counted after 48 h of incubation at 28 ± 2°C. The CFU was calculated as:

Colony⁢forming⁢unit⁢(CFU)=Number⁢of⁢colonies×dilution⁢factor/volume⁢plated.

### Bacterial cell membrane injury: A confocal microscopic analysis

The fungicide-induced bacterial membrane integrity and mortality were determined by confocal laser scanning microscopy (Xia et al., 2022). In brief, bacteria culture was inoculated in MSM liquid medium supplemented with 3,000 μg/ml CBZM fungicide and incubated in a rotatory shaker for 48 h at 28°C. After the incubation of the treated and un-treated cultures, the pellet was collected by centrifugation and 2–3 times washed with PBS (phosphate buffer saline). In 100 μl of fungicide treated and un-treated bacterial suspension, 5 μl of propidium iodide (PI) and 5 μl of acridine orange (AO) were added and incubated for 10 min at 28 ± 2°C. Then the samples were centrifuged at 5,000 rpm for 10 min to remove the unbound dyes, and the pellets were resuspended in PBS buffer. Samples were transferred to glass slides and observed under confocal laser scanning microscope (CLSM) (Leica Confocal Microscope, Germany).

### Effect of fungicide on plant growth-promoting attributes of the rhizobacteria

All the PGP activities of CBZM fungicide treated and un-treated rhizobacteria were assayed by using a standard method as given in the below section.

#### Indole acetic acid production

The indole acetic acid (IAA) production of treated and untreated bacterial cultures was determined by following the method of Bric et al. (1991). In 2 ml of cell-free rhizobacterial culture, perchloric acid and Salkowski reagent were added and incubated for 30 min at 28°C under dark conditions. IAA was quantified by taking absorbance at 530 nm using a UV-VIS spectrophotometer (Shimadzu, Model UV-1601) spectrophotometer and the amount of IAA production was calculated by using the standard curve of IAA (Bano and Musarrat, 2003).

#### Phosphate solubilization

Phosphate (P) solubilization activity was tested on National Botanical Research Institute Phosphate (NBRIP) medium (Mehta and Nautiyal, 2001). Fungicide treated and un-treated rhizobacterial cultures were spot inoculated on NBRIP agar plates and incubated for 24–96 h at 28 ± 2°C. The appearance of a clear halo zone around the bacterial growth indicated the positive results of P-solubilization. Quantitative estimation of P solubilization was performed by the method described by Fiske and Subbarow (1925) using NBRIP broth (Mehta and Nautiyal, 2001).

#### Activity of ACC deaminase

Activity of ACC deaminase (ACCD) activity was analyzed by following the method of Penrose and Glick (2003). Rhizobacterial cultures were grown in a DF salt minimal medium supplemented with 3 mM ACC or 0.1 M (NH_4_)_2_SO_4_ as the sole nitrogen source. After incubation at 28 ± 2°C for 96 h, the bacterial growth on the DF salt minimal agar plate amended with 3 mM ACC showed positive results for ACC deaminase activity. For quantitative estimation of ACCD activity, rhizobacterial cultures grown in liquid minimal salt medium with or without 3 mM ACC were centrifuged, and collected cells were washed 2–3 times with Tris–HCl (0.1 M, pH 7.5), followed by in cells, 1 ml of Tris–HCl (0.1 M, pH 8.5) was added. A further 5% toluene solution was added for labilization.

#### Siderophore production

Siderophore production efficiency was assessed by spot inoculation of freshly grown rhizobacterial culture on a CAS (Chrome azurol S) agar medium plate. After the incubation at 28 ± 2°C for 24–72 h, the appearance of a yellow-to-orange color zone around the rhizobacteria colony is an indication of positive results of the siderophore production (Schwyn and Neilands, 1987) method. Through the CAS-shuttle assay (Payne, 1994), quantitative estimation of siderophore production was determined in MM9 medium (amended glucose 1% w/v). The loss of blue color of the reaction mixture due to the removal of the iron from the dye complex indicates the siderophore present in the supernatant. The quantity of siderophore production was determined by taking OD at 630 nm and quantifying it using the formula:

%Unitofsiderophore=Ar-As/Ar× 100

where Ar denotes the reference (CAS reagent) OD at 630 nm and As denotes the test sample OD at 630 nm.

#### Ammonia production

The ammonia production of fungicide treated and un-treated cultures was compared by following the standard protocol of Cappuccino and Sherman (1992). A total of 20 μl of actively grown rhizobacterial culture was inoculated in 10 ml of freshly prepared peptone water and shaken for 24–48 h at 28 ± 2°C followed by the addition of 0.5 ml of Nessler’s reagent. The appearance of a dark yellow to brown color indicates positive ammonia production.

### Protein estimation

The total protein content of fungicide treated and un-treated rhizobacterial cells was estimated by following the standard method of Bradford (1976). The amount of protein in the rhizobacterial cells was determined spectrophotometrically at 595 nm, and protein content was calculated by using the standard curve of bovine serum albumin (BSA).

### Determination of lipid peroxidation

The lipid peroxidation (LPO) was analyzed by measuring the end product malondialdehyde (MDA), according to the method of Heath and Packer (1968). Finally, the optical density of the obtained supernatant was measured at 532 nm (all lipids) and 600 nm (all lipids except for MDA), and the amount of MDA was expressed in nmol/ml.

### Antioxidant enzymes assay

Freshly grown fungicide-treated and un-treated rhizobacterial cultures were centrifuged at 10,000 rpm for 10 min, and cell pellets were harvested. In the collected cell pellet, Tris–HCl was added to obtain a homogenous bacterial suspension. Furthermore, rhizobacterial cell suspensions were sonicated, and then sonicated cell suspensions were centrifuged (at 12,000 rpm for 10 min) to eliminate the cell debris. The supernatant containing cellular extract was used for the assay of antioxidant enzyme activity (Srivastava et al., 2017). Antioxidant enzymes like superoxide dismutase (SOD) (ADS-W-KY011), catalase (CAT) (ADS-W-KY002), glutathione peroxidase (GPX) (ADS-W-G003), ascorbate peroxidase (APX) (ADS-W-VC005), and glutathione reductase (GR) (ADS-W-FM029), activities were measured using an enzyme-linked immune sorbent assay (ELISA) kit (Jiangsu, China) by following the manufacturer’s protocol.

### Statistical analysis

Data obtained from this study are the mean of three individual replications ± standard error. SPSS (ver. 16.0) statistical software (IBM, Armonk, NY, USA) was used to analyze the data through ANOVA (analysis of variance) and DMRT (Duncan’s multiple range test) at *p* ≤ 0.05.

## Results and discussion

### Isolation and characterization of the rhizobacterial isolate

In the present study, a total of 18 dominant rhizobacterial colonies, i.e., from ANCB-1 to ANCB-18, were obtained from the collected rhizosphere soil sample of sugarcane on MSA medium plate supplemented with 500 μg/ml carbendazim. On the basis of the initial fungicide stress and plant growth promoting traits assay, a multiple PGPR bacterial isolate ANCB-12, which showed its growth at a higher carbendazim concentration, was selected for further study. The selected isolate ANCB-12 was subjected to phenotypic, biochemical, and metabolic characterization. The obtained rhizobacterial strain ANCB-12 was a Gram-positive, motile, rod-shaped, endospore-forming rhizobacteria based on phenotypic characteristics. The biochemical characteristics demonstrated that isolate ANCB-12 showed positive results for citrate utilization, catalase, methyl red, oxidase, indole, lysine utilization, nitrate reduction, and ornithine utilization but was found negative for the Ortho-Nitrophenyl-β-galactoside (ONGC), Voges–Proskauer test, arginine, phenylalanine deaminase, and H_2_S production (Table 1). The great metabolic flexibility of the bacterial isolate allows them to inhabit variable environments such as those reported in the present study. Similarly, by referring to Bergey’s Manual of Systematic Bacteriology Al-Enazi et al. (2022) characterized pesticide-tolerant *Pseudomonas* sp. from the rhizosphere soil of *Vigna radiata* (L.).

**TABLE 1:** Morphological and biochemical characterization of the rhizobacteria ANCB-12.

Tests	ANCB-12
Gram reaction	+
Shape	R
pH	6.0–8.5
NaCl tolerance	Up to 8%
Motility	+
Endospore	+
Voges–Proskauer’s	−
Citrate	+
Methyl red	+
ONPG	−
Nitrate reduction	+
Catalase	+
Oxidase	+
Arginine	−
Lysine utilization	+
Ornithine utilization	+
Phenylalanine deamination	−
H2S production	−

### Carbon utilization pattern

Soil microorganisms require some basic nutrients (such as carbon and nitrogen) for their growth and survival and to maintain their metabolic functions. Thus, microorganisms’ ferment or metabolize a wide range of simple sugars, complex carbohydrates, and amino acids for their growth and energy. The utilization patterns of carbon and amino acid sources may reveal information about the microbial biochemical pathways for various biological activities as well as for comparing the various microorganisms. With this account, in the present study, the metabolic profile through the C-utilization pattern of the isolate ANCB-12 was analyzed through “BIOLOG phenotype micro-array™ GNIII-carbon plate.” The results of the BIOLOG assay revealed that isolate ANCB-12 was positive for 19 sugars, 2 reducing sugars, chemically sensitive for 13 substrates, 6 amino acids, 7 hexose-PO_4_, 7 hexose acids, 10 carboxylic acids, esters, and fatty acids (Supplementary Table 1). The results of carbon utilization through BIOLOG assay showed that the tested rhizobacterial isolate was metabolically active. The results are in line with the study of Li et al. (2017) and Singh et al. (2020) who used BIOLOG based C-utilization assay to the exploration of metabolically active rhizobacteria of sugarcane.

### Molecular identification by phylogenetic analysis

**FIGURE 1:**
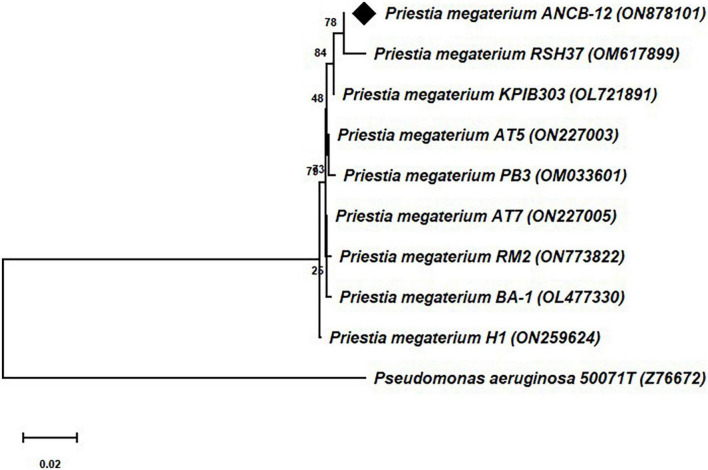
Phylogenetic tree of 16S rRNA gene sequence of the isolate ANCB-12 with similar bacterial sequences of the NCBI database. The tree was constructed by using the neighbor joining method with 1,000 bootstrap re-samplings. *Pseudomonas aeruginosa* was taken as an out group.

Molecular identification of the rhizobacteria was carried out through structural 16S rRNA gene sequencing analysis. The comparison of the obtained nearly ∼1.5 kb 16S rRNA gene sequences through BLAST search with the available bacterial sequences in the NCBI Genbank public databases indicates that the isolate ANCB-12 is closely related to *Priestia megaterium* with 100% sequence similarity. The obtained 16S rRNA gene nucleotide sequence of the rhizobacterial isolate ANCB-12 was submitted to GenBank with accession number ON878101. Figure 1 shows a phylogenetic tree made from the isolate ANCB-12 and similar bacterial 16S RNA gene sequences of NCBI database. The tree was made using the neighbor-joining method and 1,000 bootstrap samples.

16S rRNA gene sequencing analysis is one of the most widely used methods for bacterial identification and phylogeny/taxonomy analysis (Jamali et al., 2020). In accordance with our results, Sharma et al. (2019, 2021) have also isolated rhizospheric bacteria from rhizospheres of chickpea and sugarcane from different agro-climatic regions, and molecularly identified them through 16S RNA partial gene sequencing analysis.

### Rhizobacterial growth kinetics and viability analysis

**FIGURE 2:**
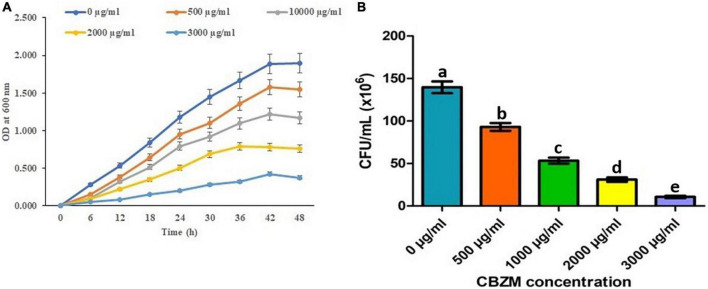
Effect of different concentrations of CBZM on rhizobacteria ANCB-12 growth **(A)** and variability in terms of CFU count **(B)** up to 48 h time duration. The data represent the mean ± SEM (*n* = 3) of three replicates. The error bars display the standard error mean (SEM). a–e indicate the statistically significant value. Letters on each bar denote significant variances (*p* ≤ 0.05) according to the DMRT test.

To control the plants’ diseases and to enhance agriculture production, an overdose of pesticides and fungicides imposes a negative effect on non-targeted beneficial microorganisms. Thus, in the present study, the effects of carbendazim fungicide on the growth of 18 isolated rhizobacterial (ANCB-1 to ANCB-18) were evaluated. At the initial screening level, all 18 isolates were spot inoculated on the MSA medium plates stressed by adding different concentrations of fungicide ranging from 0, 0.05% (500 μg/ml), 0.1% (1,000 μg/ml, recommended dose), 0.2% (2,000 μg/ml), and 0.3% (3,000 μg/ml). After the incubation time, results showed that all the tested rhizobacteria did not show growth. Only one isolate, ANCB-12, was found to be significantly tolerant to higher fungicide stress, so isolate ANCB-12 was chosen for a growth kinetic study in a liquid medium with varying concentrations of carbendazim (Figure 2A). Generally, pesticides can change the growth of tolerant or degrading bacteria (Peters et al., 2014). In the present study, the growth of the isolate ANCB-12 revealed a distinct pattern of growth when exposed to increasing concentrations of fungicide. Results in the form of a growth curve showed that in an un-treated medium, the isolate revealed luxurious growth (Figure 2A), while as the concentration of fungicide increased, the growth was sluggish as an adaptation period and a long lag phase was observed before reaching exponential growth. Isolate tolerates up to 0.25% fungicide concentration, whereas compared to the lower fungicide concentration, the higher concentration had an adverse toxic effect on bacterial life. Results showed the maximum concentration (0.3%) of fungicide has a killing effect on bacterial cell CFU. Lower growth and CFU with increasing concentration of fungicide may be due to the intake of fungicide, which influences the damage of the cell membrane and metabolic process of the bacteria (Figure 2B; Reshma et al., 2018). Tolerance to fungicides is assumed to be a distinctive characteristic among soil-living beneficial microorganisms, which are led by unique physiological, metabolic, and genetic features (Khan et al., 2020). In 2012, from the mustard rhizosphere, Ahemad and Khan isolated *Pseudomonas putida* PS9 which exhibited a different level of tolerance to various fungicides ranging from 1,400 to 3,200 μg ml^–1^ concentrations. Similarly, a *Bacillus subtilis* BC8 strain isolated from the cabbage rhizosphere soil showed a different growth pattern under different concentrations of pesticides and fungicides ranging from 0 to 3,200 μg ml^–1^ (Shahid et al., 2019). The results of the current study are also in accordance with the observation of Nagaraju et al. (2017), who reported the lethal effect of fungicides, herbicides, and zinc solubilizing bacteria.

### Impact of fungicide on rhizobacterial cell membrane

**FIGURE 3:**
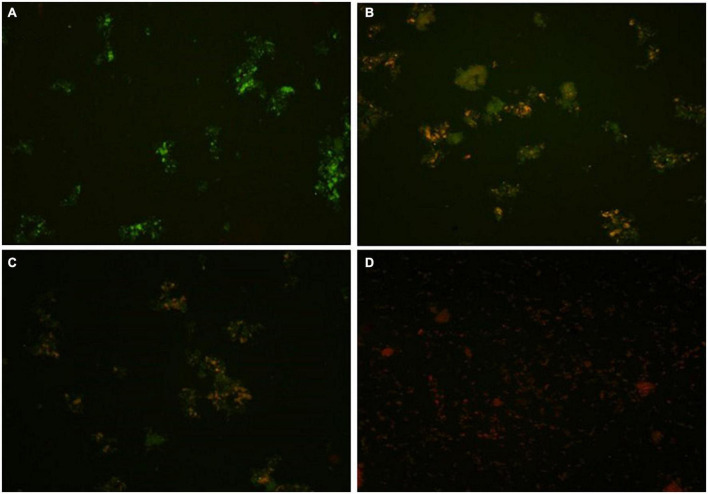
Confocal laser scanning microscopy (CLSM) based membrane permeability analysis of isolate ANCB-12 under different CBZM concentrations. **(A)** Untreated cell, **(B)** 1,000 μg/ml CBZM treated cell, **(C)** 2,000 μg/ml CBZM treated cell, and **(D)** 3,000 μg/ml CBZM treated cell.

In the present study, an advanced confocal laser scanning microscopy (CLSM) technique was used to analyze the toxic effects of the fungicide CBZM on rhizobacterial membrane permeability. Results of CLSM analysis showed that as the fungicide treatment level increased, a huge number of red fluorescence releasing rhizobacterial cells were observed (Figure 3). This indicated the highly toxic effect on the viability of rhizobacterial cells, which led to cell damage over controlled un-treated cells (Figure 3). However, no red-colored cells were detected in CLSM images of control CBZM un-treated cells (Figure 3A). This is because toxic fungicides increase cell permeability, allowing dyes to enter the cell and bind with intracellular components and nucleic acids. The results of the present study are in line with the study of Kudoyarova et al. (2017), who differentiated between dead and live cells of *B. subtilis* strain BC8 in CLSM analysis. Similar to our results, similar observations were obtained in the studies of Shahid et al. (2019; 2021a,b), who reported an increasing number of dead cells of *Bradyrhizobium japonicum, Pseudomonas* sp., and *Enterobacter cloacae* after treatment with increasing concentrations of fungicides.

### Impact of carbendazim stress on plant growth promoting attributes of ANCB-12 isolate

#### Indole-3-acetic acids

**FIGURE 4:**
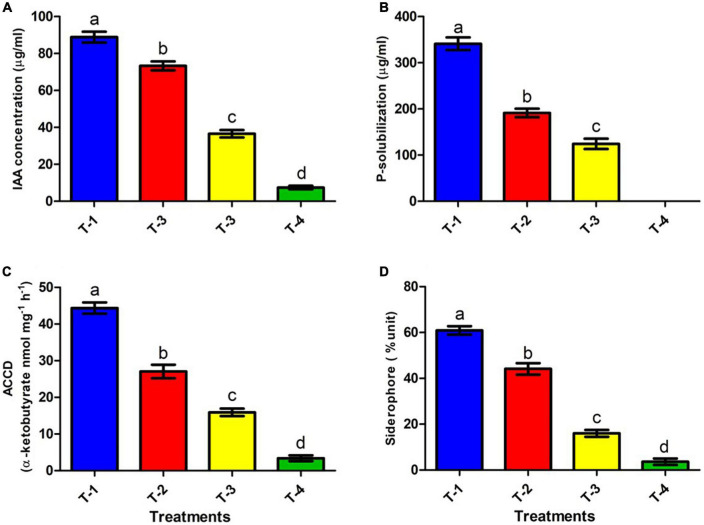
Effect of CBZM stress on plant growth promoting attributes of rhizobacteria isolates ANCB-12. (T-1) No CBZM concentration, (T-2) 1,000 μg/ml CBZM, (T-3) 2,000 μg/ml CBZM, and (T-4) 3,000 μg/ml CBZM. **(A)** IAA production, **(B)** phosphate solubilization, **(C)** ACC deaminase activity and **(D)** siderophore production. The data represent the mean ± SEM (*n* = 3) of three replicates. The error bars display the standard error mean (SEM). Letters on each bar denote significant variances (*p* ≤ 0.05) according to the DMRT test.

Plant growth promoting rhizobacteria producing IAA plays an imperative role in cell division and differentiation, especially in root development and overall plant growth (Grover et al., 2021). We analyzed the IAA producing ability of ANCB-12 under CBZM treatment conditions. Under controlled condition (in the absence of fungicide), isolate ANCB-12 secreted a considerable amount of IAA, 88.8 μg/ml (T-1). However, it was noticed that the IAA production was significantly (*p* ≤ 0.05) reduced with increasing concentrations of fungicide. Results showed that the increasing concentrations of fungicide reduced the synthesis of IAA by 17.5, 58.8, and maximally 91.6% at 1,000 μg/ml (T-2), 2,000 μg/ml (T-3), and 3,000 μg/ml (T-4) fungicide levels, respectively, over the un-treated control (Figure 4A). Higher dosage of pesticides may bind with cellular molecules and affect the metabolism, which may be the cause of reduced IAA. A similar result was observed in *Paenibacillus* sp. treated with organochlorine pesticides (Mundi et al., 2020).

#### Phosphate solubilization

Phosphate solubilization is another important feature of soil bacteria, by which soil microbes provide the soluble form of phosphate to the plant. The release of low molecular weight organic acids by phosphate solubilizing bacteria (PSB) converts insoluble phosphate to soluble phosphate (Alori et al., 2017). In the present study, quantitative estimation of phosphate-solubilization of ANCB-12 isolate under graded concentrations of carbendazim was evaluated by using the phosphate-solubilizing specific standardized NBRIP liquid medium (Figure 4B). Results of quantitative estimation revealed that a considerable amount of TCP (340.9 μg/ml) was solubilized in fungicide deficient liquid medium (controlled condition: T-1) by the rhizobacterial isolate ANCB-12. Results showed that with increasing concentration of CBZM, i.e., at 1,000 μg/ml (T-2) and 2,000 μg/ml (T-3) a decline of P-solubilizing activity by 43.8 and 63.5%, respectively, was observed over control (T-1) (Figure 4B). However, at a higher 3,000 μg/ml (T-4) CBZM concentration, there was no P-solubilizing activity observed. The results are in contrast with other findings of earlier research where *Pseudomonas fluorescens* showed a declining trend of TCP solubilization under pesticide treatment (Al-Enazi et al., 2022).

#### ACC deaminase activity

The rhizobacterial ACC deaminase enzyme is a pyridoxal phosphate dependent enzyme, which by reducing ethylene content, protect and promotes plant growth under various biotic and abiotic conditions including under fungicide stress. (Singh et al., 2022). The isolate *P. megaterium* ANCB-12, grown under control conditions, produced a significant amount of ACC deaminase by converting 44.3 α-ketobutyrate nmol mg^–1^ protein h^–1^, similar to IAA production. However, the amount of ACCD was reduced with an increasing concentration of fungicide (Figure 4C). Results showed that the amount of ACCD was reduced by 38.9, 64.1, and a maximum of 92.3 at 1,000 μg/ml (T-2), 2,000 μg/ml (T-3), and 3,000 μg/ml (T-4) fungicide levels, respectively, over unstressed control (T-1). Several studies (Syed et al., 2021; Al-Enazi et al., 2022) have found that fungicides and heavy metals reduce the ACC deaminase activity of soil bacteria in a similar way.

#### Siderophore production

Iron is an essential molecule for a plant’s chlorophyll generation, photosynthesis, and development of resistance against pathogens. While in bacteria, iron is crucial for physiology, metabolism, DNA replication, regulatory proteins, transcription, energy production, and plant microbial interaction. A deficiency of iron results in the disturbance of all these processes. Under such a situation, PGPR produces low-molecular-weight substances called siderophores that efficiently chelate the ferric ion (Fe^3+)^ and act as carriers for the entry of Fe (III) into the cell (Ferreira et al., 2019). In the present study, the siderophore producing ability of isolate ANCB-12 was determined on CAS broth amended with variable rates of fungicide (Figure 4D). Results showed that, like IAA and P-solubilizing activities, the capacity of the isolate ANCB-12 for siderophore production at normal controlled conditions (T-1) was 60.9%. Furthermore, results showed that the amount of siderophore production was gradually decreased by 28.2, 73.9, and 94.1% at 1,000 μg/ml (T-2), 2,000 μg/ml (T-3), and 3,000 μg/ml (T-4) fungicide levels, respectively, over the un-treated control (Figure 4D). The results of the present study are in accordance with the observation of Kumar et al. (2019), who also observed a declining trend of siderophore under pesticide treatment.

#### Ammonia production

Ammonia production by PGPR is beneficial for supplying nitrogen to their host plants and promoting growth (Marques et al., 2010; dos Santos et al., 2020). In our study, *P. megaterium* ANCB-12’s ability to make ammonia was not much affected by the higher levels of fungicide.

### Lipid peroxidation (LPO)

**FIGURE 5:**
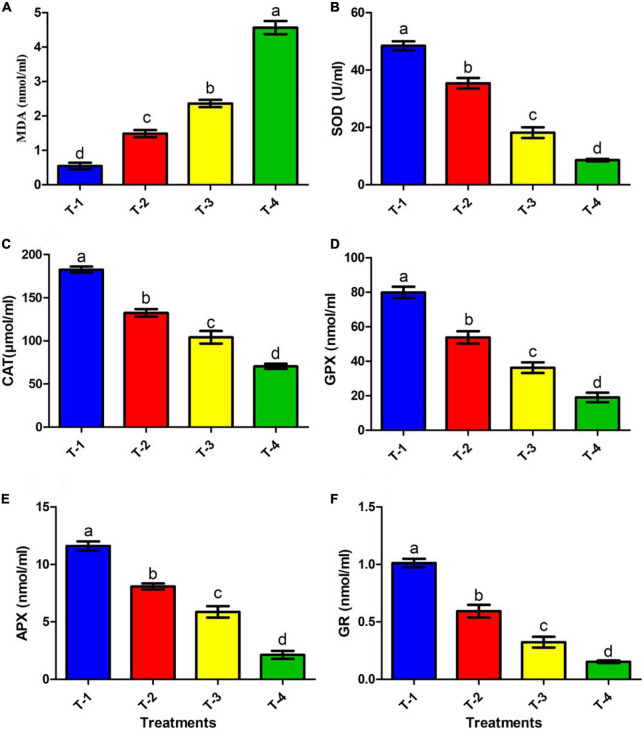
Effect of CBZM stress on antioxidant enzyme activities of rhizobacteria isolates ANCB-12. (T-1) No CBZM concentration, (T-2) 1,000 μg/ml CBZM, (T-3) 2,000 μg/ml CBZM, and (T-4) 3,000 μg/ml CBZM. **(A)** MDA content, **(B)** SOD activity, **(C)** CAT activity, **(D)** GPX activity, **(E)** APX activity and **(F)** GR activity. The data represent the mean ± SEM (*n* = 3) of three replicates. The error bars display the standard error mean (SEM). Letters on each bar denote significant variances (*p* ≤ 0.05) according to the DMRT test.

Bacterial membrane integrity in terms of LPO due to stress conditions is a major stress marker (Heath and Packer, 1968). Therefore, considering this fact, in the present study, the membrane integrity of the isolate ANCB-12 under fungicide CBZM stress was further analyzed by MDA content. Results showed that MDA levels of the isolate ANCB-12 was 0.54 nmol/ml under control conditions (T-1) without any fungicide stress (Figure 5A). However, with increasing fungicide concentration, the cellular toxicity increased, which was evident by increasing MDA levels (Figure 5A). CBZM stress showed severe cellular toxicity and MDA content was increased to 1.48, 2.36, and 4.56 nmol/ml at 1,000 (T-2), 2,000 (T-3), and 3,000 μg/ml (T-4) CBZM concentration treatments, respectively. Results demonstrated that the levels nearly doubled with increasing fungicide treatments (*p* < 0.05; Figure 5A). The generation of MDA is evidence of oxidative stress within the cells. The findings are consistent with those of Shahid and Khan (2018), who found an increase in MDA levels in *B. subtilis* under CBZM and kitazin stress. In a recent study, Rovida et al. (2021) also found that herbicide-treated *Pseudomonas* sp. had a higher level of MDA.

### Effect of carbendazim on antioxidant enzymes activities

Antioxidant enzymes such as SOD, CAT, GPX, and GR have been found in almost all cellular compartments and play a significant role in the neutralization or detoxification of toxic ROS molecules. SOD is an enzyme that is activated by ROS generation and the breakdown of singlet oxygen radical (^1^O_2_^–^) or superoxide radical (*O_2_) into H_2_O_2_ and O_2_ (Hasanuzzaman et al., 2020). However, this SOD induced end product H_2_O_2_ retains its potential to induce cell damage by conversion of H_2_O_2_ into hydroxyl radical (*OH). Thus subsequently, CAT, APX, and GPX detoxifies the H_2_O_2_ into H_2_O and O_2_, as well as inhibited LPO and protect cell damage (Sharma et al., 2012; Ighodaro and Akinloye, 2018). On the other hand, GR kept the intracellular glutathione pool (GSH) in a reduced state, which acts as a direct and indirect antioxidant for scavenging ROS. In the present study, we assessed the effects of different doses of fungicide CBZM on the activities of antioxidant enzymes, which are directly related to the detoxification of ROS molecules (Figure 5). Results showed that the addition of CBZM causes oxidative stress in the ANCB-12 rhizobacterial strain. SOD activity under non-stress conditions (0 μg/ml: T-1) was maximal (48.4 U/ml). CBZM stress gradually reduces SOD activity by 26.9, 62.5, and 82.3% at the recommended doses of T-2 (1,000 μg/ml), T-3 (double dose: 2,000 μg/ml), and T-4 (triple dose: 3,000 μg/ml) levels of CBZM (Figure 5B). Similarly, CAT activity was highest at normal conditions, i.e., 182.6 μmol/ml, which was significantly decreased by 27.4, 42.9, and 61.4% when exposing them to T-2, T-3, and T-4 CBZM stress concentrations, respectively, over non-CBZM stressed control (Figure 5C). Similar to SOD and CAT activities, GPX activity also decreased with an increasing dose of fungicide. Results showed that GPX activity maximally declined by 76.1% when exclusively exposed to the highest stress of CBZM (T-4: 3,000 μg/ml), followed by 54.5 and 32.6% at T-3 and T-4 CBZM stress levels, respectively, over control (T-1; 0 μg/ml CBZM) (Figure 5D). Furthermore, similar trends were also observed in APX activity, where activity maximally decreased by 81.6% at T-4 treatment of CBZM concentration, followed by 49.3 and 30.3% decreases under T-3 and T-2 CBZM stress conditions, respectively, over the non-stress control (T-1) condition (Figure 5E). In addition, results showed that the GR activity of bacterial cells decreased by 84.8, 68.0, and 41.3% at T-4, T-3, and T-2 CBZM stress conditions, respectively, as compared to the T-1 non-stress level of CBZM (Figure 5F). Overall, the results of the present study showed that with increasing fungicide stress, all the ROS scavenging antioxidants SOD, CAT, GPX, APX, and GR enzyme activity were gradually downregulated. The decrease in the activities of these enzymes is related to the increase in the free movement of H_2_O_2_, leading to increased cell permeability, LPO (MDA), and disruption of nuclear materials, which causes direct damage to the cell. These results are directly related to our growth kinetics, CLMS analysis, and increased level of LPO (MDA) under a gradient dose of fungicide. Similar damaging effects on antioxidant enzymes were observed with an increasing dose of pesticide stress by Shahid and Khan (2018). Our observations are also supported by similar results in *Pseudomonas* spp., *Enterobacter asburiae*, and *Mesorhizobium ciceri* under different pesticide stress conditions (Martins et al., 2011; Rovida et al., 2021; Shahid et al., 2021a).

### Heatmap of Pearson’s correlation analysis

**FIGURE 6:**
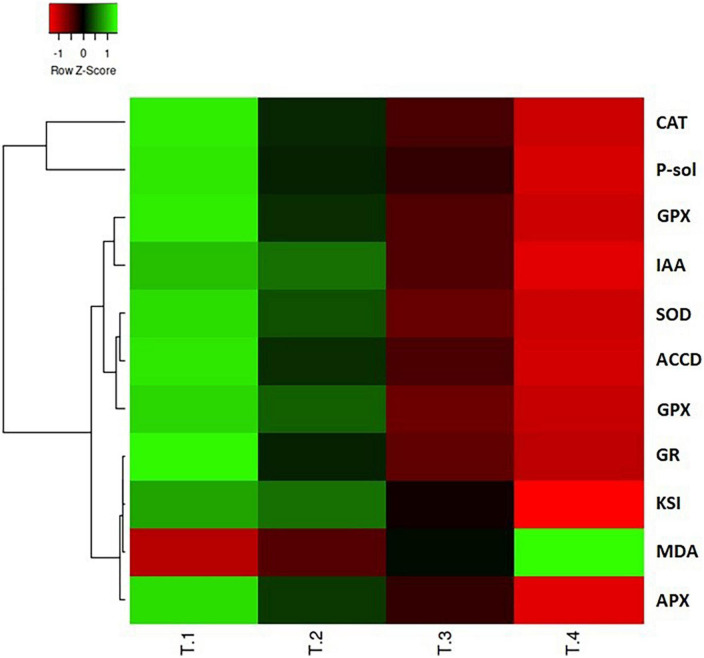
Heatmap multivariate data analysis representing the responses of various variables of PGP attributes and antioxidant enzyme defense system against the different treatments of CBZM applied. T-1: control un-treated cell; T-2: recommended 1,000 μg/ml CBZM; T-3: double dose, i.e., 2,000 μg/ml CBZM; and T-4: triple dose, i.e., 3,000 μg/ml CBZM. IAA, indole acetic acid; P-sol, phosphate solubilization; ACCD, ACC deaminase; Sid, siderophore; KSI, phosphate solubilization index; CAT, catalase; SOD, superoxide dismutase; GPX, glutathione peroxidase; APX, ascorbate peroxidase, polyphenol oxidase; GR, glutathione reductase; MDA, malondialdehyde.

Heatmap analysis revealed that the plant growth promoting attributes, LPO, and antioxidant enzyme variables displayed differential responses in the integrated heatmap against the various dosage of carbendazim fungicide treatments (Figure 6). The results of heatmap multivariance analysis clearly demonstrate that CBZM stress imposed adverse effects on all the tested functional and metabolic attributes of the beneficial rhizobacteria.

## Conclusion

In conclusion, the results of the present study stated that an overdose of CBZM significantly reduced the growth of a PGPR strain *P. megaterium* ANCB-12, cell membrane disintegration, and beneficial plant growth promoting metabolic substances. Furthermore, increasing the concentration of the fungicide CBZM altered the defense system of ROS scavenging antioxidant enzymes, which is essential for the survival of bacterial cells under stress conditions. These findings pointed out the negative effect of high dosage of CBZM on plant beneficial bacteria present into the soil. Along with this, an eco-friendly alternative such as biofungicide needs to be developed and use in the integrative pest management (IPM) model for sustainable agriculture production.

## Data availability statement

The datasets presented in this study can be found in online repositories. The names of the repository/repositories and accession number(s) can be found in the article/Supplementary material.

## Author contributions

AS and Y-RL conceptualized the idea and designed the experiments. AS executed all the laboratory experiments, analyzed the data, and wrote the manuscript. X-PS contributed to resource management. AV, RS, PS, and D-JG contributed to software and necessary material. AV, SG, KV, and Y-RL critically revised the manuscript. All authors read and agreed to the published version of the manuscript.
