# Supplementary material for: Impact of carbendazim on cellular growth, defence system and plant growth promoting traits of Priestia megaterium ANCB-12 isolated from sugarcane rhizosphere
Source: Front Microbiol. 2022 Dec 20;13:1005942. doi: 10.3389/fmicb.2022.1005942 (PMC9808048; doi:10.3389/fmicb.2022.1005942)
Supplement: Supplementary file 1 [file Table_1.DOCX]

**Supplementary Table ST-1**

| **S. No.** | **Serial No. (Biolog)** | **Substrate** | **Chemical Guild** | **ANCB-12** |  | **S. No.** | **Serial No. (Biolog)** | **Substrate** | **Chemical Guild** | **ANCB-12** |
| --- | --- | --- | --- | --- | --- | --- | --- | --- | --- | --- |
| 1 | A1 | Negative Control | Sugars | - |  | 25 | C1 | α-D-Glucose | Sugars | **-** |
| 2 | A2 | Dextrin | Sugars | - |  | 26 | C2 | D-Mannose | Sugars | **+** |
| 3 | A3 | D-Maltose | Sugars | - |  | 27 | C3 | D-Fructose | Sugars | **+** |
| 4 | A4 | D-Trehalose | Sugars | + |  | 28 | C4 | D-Galactose | Sugars | + |
| 5 | A5 | D-Cellobiose | Sugars | **+** |  | 29 | C5 | 3-Methyl Glucose | Sugars | + |
| 6 | A6 | Gentiobiose | Sugars | + |  | 30 | C6 | D-Fucose | Sugars | - |
| 7 | A7 | Sucrose | Sugars | + |  | 31 | C7 | L-Fucose | Sugars | + |
| 8 | A8 | D-Turanose | Sugars | - |  | 32 | C8 | L-Rhamnose | Sugars | + |
| 9 | A9 | Stachyose | Sugars | + |  | 33 | C9 | Inosine | Sugars | **+** |
| 10 | A10 | Positive Control | Chemical Sensivity | **+** |  | 34 | C10 | 1% Sodium Lactate | Lactic acid | **-** |
| 11 | A11 | pH 6 | Acidic pH | **+** |  | 35 | C11 | Fusidic Acid | Chemical Sensivity | **-** |
| 12 | A12 | pH 5 | Acidic pH | **-** |  | 36 | C12 | D-Serine | Chemical Sensivity | **+** |
| 13 | B1 | D-Raffinose | Sugars | + |  | 37 | D1 | D-Sorbitol | Chemical Sensivity | - |
| 14 | B2 | α-D-Lactose | Sugars | + |  | 38 | D2 | D-Mannitol | Chemical Sensivity | **+** |
| 15 | B3 | D-Melibiose | Sugars | + |  | 39 | D3 | D-Arabitol | Chemical Sensivity | **-** |
| 16 | B4 | β-Methyl-D-Glucoside | Sugars | + |  | 40 | D4 | myo-Inositol | Chemical Sensivity | + |
| 17 | B5 | D-Salicin | Sugars | **+** |  | 41 | D5 | Glycerol | Chemical Sensivity | **+** |
| 18 | B6 | N-Acetyl-D-Glucosamine | Sugars | + |  | 42 | D6 | D-Glucose-6-PO_4_ | Hexose-PO_4_ | **+** |
| 19 | B7 | N-Acetyl-β-DMannosamine | Sugars | + |  | 43 | D7 | D-Fructose-6-PO_4_ | Hexose-PO_4_ | **+** |
| 20 | B8 | N-Acetyl-D-Galactosamine | Sugars | **-** |  | 44 | D8 | D-Aspartic Acid | Chemical Sensivity | + |
| 21 | B9 | N-AcetylNeuraminic Acid | Sugars | - |  | 45 | D9 | D-Serine | Chemical Sensivity | + |
| 22 | B10 | 1% NaCl | Sodium Chloride | **+** |  | 46 | D10 | Troleandomycin | Chemical Sensivity | **-** |
| 23 | B11 | 4% NaCl | Sodium Chloride | **+** |  | 47 | D11 | Rifamycin SV | Chemical Sensivity | **+** |
| 24 | B12 | 8% NaCl | Sodium Chloride | **+** |  | 48 | D12 | Minocycline | Chemical Sensivity | **-** |

**Continue Supplementary Table ST-1….**

| **S. No.** | **Serial No. (Biolog)** | **Substrate** | **Chemical Guild** | **ANCB-12** |  | **S. No.** | **Serial No. (Biolog)** | **Substrate** | **Chemical Guild** | **ANCB-12** |
| --- | --- | --- | --- | --- | --- | --- | --- | --- | --- | --- |
| 49 | E1 | Gelatin | Amino acid | **+** |  | 73 | G1 | p-Hydroxy- Phenylacetic Acid | Carboxylic acids, esters and fatty acids | **-** |
| 50 | E2 | Glycyl-L-Proline | Amino acid | **+** |  | 74 | G2 | Methyl Pyruvate | Carboxylic acids, esters and fatty acids | - |
| 51 | E3 | L-Alanine | Amino acid | **+** |  | 75 | G3 | D-Lactic Acid Methyl Ester | Carboxylic acids, esters and fatty acids | - |
| 52 | E4 | L-Arginine | Amino acid | **+** |  | 76 | G4 | L-Lactic Acid | Carboxylic acids, esters and fatty acids | **+** |
| 53 | E5 | L-Aspartic Acid | Amino acid | **-** |  | 77 | G5 | Citric Acid | Carboxylic acids, esters and fatty acids | **+** |
| 54 | E6 | L-Glutamic Acid | Amino acid | **-** |  | 78 | G6 | α-Keto-Glutaric Acid | Carboxylic acids, esters and fatty acids | **-** |
| 55 | E7 | L-Histidine | Amino acid | **+** |  | 79 | G7 | D-Malic Acid | Carboxylic acids, esters and fatty acids | **+** |
| 56 | E8 | L-Pyroglutamic Acid | Amino acid | **+** |  | 80 | G8 | L-Malic Acid | Carboxylic acids, esters and fatty acids | **+** |
| 57 | E9 | L-Serine | Amino acid | **-** |  | 81 | G9 | Bromo-Succinic Acid | Carboxylic acids, esters and fatty acids | + |
| 58 | E10 | Lincomycin | Chemical Sensivity | **+** |  | 82 | G10 | Nalidixic Acid | Chemical Sensivity | **+** |
| 59 | E11 | Guanidine HCl | Chemical Sensivity | **-** |  | 83 | G11 | Lithium Chloride | Chemical Sensivity | **+** |
| 60 | E12 | Niaproof 4 | Chemical Sensivity | **-** |  | 84 | G12 | Potassium Tellurite | Chemical Sensivity | **-** |
| 61 | F1 | Pectin | Hexose acid | - |  | 85 | H1 | Tween 40 | Carboxylic acids, esters and fatty acids | **-** |
| 62 | F2 | D-Galacturonic Acid | Hexose acid | + |  | 86 | H2 | γ-Amino-ButryricAcid | Carboxylic acids, esters and fatty acids | **-** |
| 63 | F3 | L-Galactonic Acid Lactone | Hexose acid | + |  | 87 | H3 | α-Hydroxy- Butyric Acid | Carboxylic acids, esters and fatty acids | + |
| 64 | F4 | D-Gluconic Acid | Hexose acid | **+** |  | 88 | H4 | β-Hydroxy-D,LButyricAcid | Carboxylic acids, esters and fatty acids | **-** |
| 65 | F5 | D-Glucuronic Acid | Hexose acid | + |  | 89 | H5 | α-Keto-Butyric Acid | Carboxylic acids, esters and fatty acids | + |
| 66 | F6 | Glucuronamide | Hexose acid | + |  | 90 | H6 | Acetoacetic Acid | Carboxylic acids, esters and fatty acids | + |
| 67 | F7 | Mucic Acid | Hexose acid | + |  | 91 | H7 | Propionic Acid | Carboxylic acids, esters and fatty acids | **+** |
| 68 | F8 | Quinic Acid | Hexose acid | **+** |  | 92 | H8 | Acetic Acid | Carboxylic acids, esters and fatty acids | **+** |
| 69 | F9 | D-Saccharic Acid | Hexose acid | **-** |  | 93 | H9 | Formic Acid | Carboxylic acids, esters and fatty acids | - |
| 70 | F10 | Vancomycin | Chemical Sensivity | **-** |  | 94 | H10 | Aztreonam | Chemical Sensivity | **+** |
| 71 | F11 | Tetrazolium Violet | Reducing Sugar | **+** |  | 95 | H11 | Sodium Butyrate | Chemical Sensivity | **+** |
| 72 | F12 | Tetrazolium Blue | Reducing Sugar | **+** |  | 96 | H12 | Sodium Bromate | Chemical Sensivity | **-** |

‘+’ positive for utilization; ‘-‘ negative for utilization
